# Nanoparticle Applications in Plant Biotechnology: A Comprehensive Review

**DOI:** 10.3390/plants15030364

**Published:** 2026-01-24

**Authors:** Viktor Husak, Milos Faltus, Alois Bilavcik, Stanislav Narozhnyi, Olena Bobrova

**Affiliations:** 1 Department of Biochemistry and Biotechnology, Vasyl Stefanyk Carpathian National University, Shevchenka 57, 76018 Ivano-Frankivsk, Ukraine; viktor.husak@cnu.edu.ua; 2 Czech Agrifood Research Center, Plant Physiology and Cryobiology Team, Drnovska 507/73, 16100 Prague, Czech Republic; alois.bilavcik@carc.cz; 3 Cryobiophysics Department, Institute for Problems of Cryobiology and Cryomedicine NAS of Ukraine, Pereyaslavskaya 23, 61016 Kharkiv, Ukraine

**Keywords:** green synthesis, oxidative stress, biocompatibility, genetic transformation, cryopreservation, tissue culture

## Abstract

Nanotechnology is becoming a key tool in plant biotechnology, enabling nanoparticles (NPs) to deliver biomolecules with high precision and to enhance plant and tissue resilience under stress. However, the literature remains fragmented across genetic delivery, in vitro regeneration, stress mitigation, and germplasm cryopreservation, and it still lacks standardized, comparable protocols and robust long-term safety assessments—particularly for NP use in cryogenic workflows. This review critically integrates recent advances in NP-enabled (i) genetic engineering and transformation, (ii) tissue culture and regeneration, (iii) nanofertilization and abiotic stress mitigation, and (iv) cryopreservation of plant germplasm. Across these areas, the most consistent findings indicate that NPs can facilitate targeted transport of DNA, RNA, proteins, and regulatory complexes; modulate oxidative and osmotic stress responses; and improve regeneration performance in recalcitrant species. In cryopreservation, selected nanomaterials act as multifunctional cryoprotective adjuvants by suppressing oxidative injury, stabilizing cellular membranes, and improving post-thaw viability and regrowth of sensitive tissues. At the same time, NP outcomes are highly context-dependent, with efficacy governed by dose, size, and surface chemistry; formulation; plant genotype; and interactions with culture media or vitrification solutions. Evidence of potential phytotoxicity, persistence, and biosafety risks highlights the need for harmonized reporting, mechanistic studies on NP–cell interfaces, and evaluation of environmental fate. Expected outcomes of this review include a consolidated framework linking NP properties to biological endpoints, identification of design principles for application-specific NP selection, and a set of research priorities to accelerate the safe and reproducible translation of nanotechnology into sustainable plant biotechnology and long-term germplasm preservation.

## 1. Introduction

Global food security and biodiversity conservation are increasingly interconnected challenges under mounting environmental and demographic pressures, including climate change, land degradation, and the emergence of novel plant pathogens [1,2,3,4]. Addressing these challenges requires not only improved crop performance but also the effective conservation of plant genetic resources to support future breeding, restoration, and resilience strategies [2,5].

Plant biotechnology relies on genetic transformation, in vitro regeneration, and cryopreservation to improve crops and conserve plant genetic resources; however, each of these workflows still faces major practical bottlenecks [6,7,8,9,10]. Conventional transformation and delivery approaches remain limited by host range, tissue damage, and variable reproducibility, while regeneration and cryopreservation outcomes are often constrained by genotype dependence and stress-related injury during treatment, freezing, and thawing [9,10].

In this context, nanoparticles (NPs) are increasingly explored as engineered carriers and functional additives that can support the delivery of DNA, RNA, proteins, and ribonucleoprotein complexes, and can also modulate oxidative and osmotic stress responses that influence regeneration and post-thaw recovery [11,12,13,14,15,16,17,18]. Foundational aspects of plant nanotechnology, including nanoparticle classes, delivery routes, cellular uptake mechanisms, and localization patterns in plants, have already been comprehensively reviewed elsewhere [19]. Therefore, this review provides only concise contextual background for these established concepts and instead focuses on recent advances with direct relevance to plant biotechnology outcomes, emphasizing mechanisms, reproducibility, and responsible use [16,18].

Recent studies demonstrate that, under controlled conditions, selected nanomaterials can enhance transformation efficiency, stimulate shoot regeneration or callus formation, and improve in vitro performance in recalcitrant species, sometimes acting as hormone mimics or stress modulators [14,15,16,17]. In cryopreservation, NPs have been incorporated into vitrification and encapsulation–dehydration protocols to mitigate cryoinjury and improve post-thaw recovery [18]. Despite these promising findings, most NP-based applications remain at the experimental or proof-of-concept stage, and significant knowledge gaps persist regarding long-term safety, cellular interactions, nanoparticle fate, and protocol standardization.

This review aims to critically synthesize recent advances in nanoparticle applications across four main pillars of plant biotechnology:

- Genetic transformation and molecular delivery;
- Tissue culture and plant regeneration;
- Nanofertilization and abiotic stress mitigation;
- Cryopreservation of plant germplasm.

In addition, we examine toxicological, regulatory, and environmental considerations associated with nanomaterial use in plant systems, providing a forward-looking perspective on their responsible and scalable integration into agricultural and conservation frameworks. By aligning recent developments in nanotechnology with the core methodologies of plant biotechnology, this review offers a consolidated resource for researchers seeking to enhance efficiency, reproducibility, and innovation in plant transformation, regeneration, and long-term preservation systems.

## 2. Overview of Nanoparticles in Plant Systems

Because nanoparticle types, plant delivery pathways, uptake mechanisms, and intracellular localization have been extensively reviewed elsewhere, this section is intentionally selective. We provide a brief, application-oriented overview and highlight only recent developments that are directly relevant to plant biotechnology outcomes—particularly advances in biocompatible and biodegradable formulations, surface functionalization for tissue or organelle targeting, improved colloidal stability in culture and vitrification solutions, and design features that enable reproducible, dose-controlled performance across genotypes and experimental systems.

### 2.1. Research Trends and Emerging Gaps in Plant Nanotechnology

**Figure 1:**
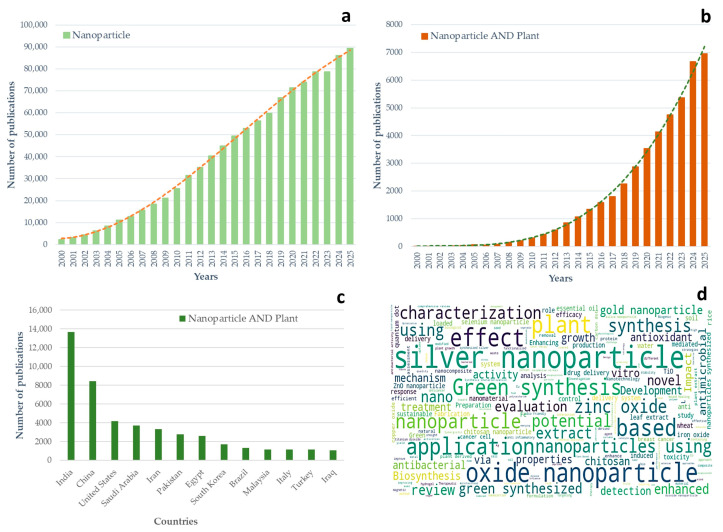
Scopus-based bibliometric overview of nanoparticle research and nanoparticle–plant studies (data retrieved on 23 December 2025). (**a**) Annual number of publications on “nanoparticle” (2000–2025); (**b**) annual number of publications indexed for the combined query “nanoparticle” AND “plant” (2000–2025); (**c**) leading countries by publication output for “nanoparticle” AND “plant”; (**d**) keyword word cloud highlighting the most frequent terms in the “nanoparticle” AND “plant” literature.

Bibliometric analysis based on Scopus records reveals a pronounced and sustained expansion of NP-related research over the past 25 years, underscoring the maturation of nanotechnology as a core scientific discipline (Figure 1a). Annual publications indexed under the keyword “nanoparticle” increased nearly 40-fold, from 2293 in 2000 to 89,389 in 2025. This trajectory reflects the transition of nanomaterials from a niche innovation to a foundational platform underpinning advances across materials science, medicine, energy, and agriculture.

When the analysis is refined to studies explicitly linking nanoparticles with plants, an even more striking pattern emerges (Figure 1b). From only eight publications in 2000, the annual output surged to 6953 publications by 2025, with a clear inflection point around 2010. This acceleration coincides with the adoption of green synthesis strategies, growing interest in nanoparticle–biological interactions, and the recognition of plants as both targets and biofactories for nanomaterials. The post-2010 growth phase highlights the increasing integration of nanotechnology into plant science, particularly in the contexts of stress physiology, nutrient delivery, and antimicrobial applications.

Geographical analysis reveals a highly uneven global distribution of research activity (Figure 1c). India emerges as the dominant contributor to nanoparticle–plant research, followed by China and the United States, reflecting strong national investments in nanoscience, agriculture, and sustainable technologies. A second tier of countries, including Saudi Arabia, Iran, and Pakistan, demonstrates substantial output, while additional contributions from Egypt, South Korea, Brazil, Malaysia, Italy, Turkey, and Iraq point to a rapidly diversifying research landscape. This distribution emphasizes the prominence of Asia and the Global South, regions where agricultural intensification, abiotic stress, and sustainable crop production represent pressing scientific and societal priorities.

The thematic structure of the field, visualized through keyword frequency analysis (Figure 1d), reveals a strong concentration around material synthesis and basic biological evaluation. Dominant terms such as “silver”, “oxide”, “green synthesis”, “biosynthesis”, “antimicrobial”, and “characterization” indicate that much of the literature focuses on metallic and metal oxide nanoparticles, particularly those produced via eco-friendly routes. While application-oriented keywords such as “plant growth”, “toxicity”, “drug delivery”, and “sustainable” reflect interdisciplinary engagement, they also suggest that many studies remain descriptive, emphasizing physiological responses rather than mechanistic or translational outcomes. Critically, the near absence of terms related to cryopreservation, germplasm conservation, and regeneration biology within this keyword landscape exposes a major conceptual and practical gap. Despite evidence that NPs can modulate oxidative stress, stabilize membranes, enhance molecular delivery, and affect heat and mass transfer, these processes are central to freezing tolerance and post-thaw recovery. However, the systematic integration of NPs into plant cryopreservation protocols remains very limited. This imbalance highlights a disconnect between the dominant research trends and some of the most pressing needs in plant biotechnology, particularly the long-term conservation of genetic resources and the recovery of recalcitrant tissues.

Taken together, Figure 1 illustrates a field characterized by rapid quantitative growth but qualitative imbalance. While NP–plant research has expanded explosively and diversified geographically, it remains heavily skewed toward synthesis-driven and short-term physiological studies. Strategic redirection toward underexplored applications, especially cryopreservation, regeneration fidelity, and long-term cellular stability, represents a critical opportunity for advancing plant nanotechnology into a more mature, solution-oriented discipline.

### 2.2. Types of Nanoparticles

Nanoparticles applied in plant biotechnology comprise a diverse suite of nanoscale materials whose physicochemical properties govern their interactions with plant tissues, modes of action, and safety profiles [19,20,21,22]. Based on structural and functional characteristics, these materials can be broadly categorized into metallic, metal oxide, carbon-based, polymeric/biogenic, and hybrid or composite NPs. Table 1 summarizes the major NP classes used in plant systems, highlighting representative examples, key properties, principal applications, and relevant references.

**Figure 2:**
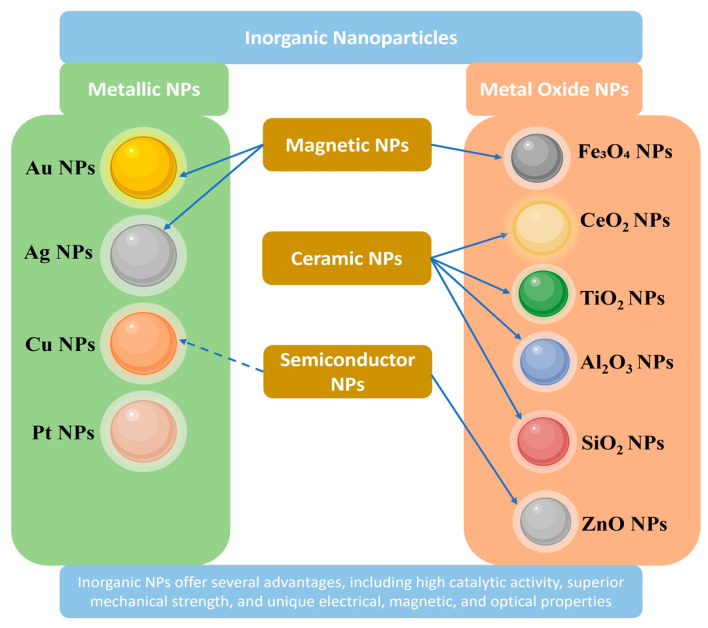
Classification of inorganic nanoparticles based on their chemical composition and functional properties. This diagram illustrates the major categories of inorganic NPs, including metallic NPs (AuNPs, AgNPs, CuNPs, PtNPs) and metal oxide NPs (e.g., Fe_3_O_4_ NPs, CeO_2_ NPs, TiO_2_ NPs, Al_2_O_3_ NPs, SiO_2_ NPs, ZnO NPs), further subclassified into magnetic, ceramic, and semiconductor NPs. The arrows indicate typical classifications, while the dashed arrow connecting Cu NPs to semiconductor NPs highlights their context-dependent classification, as Cu NPs can exhibit semiconductor-like properties under specific conditions. Color coding is used to visually distinguish nanoparticle classes and subcategories and does not imply relative importance, abundance, or functional hierarchy.

As illustrated in Figure 2, metallic nanoparticles, including silver (AgNPs), gold (AuNPs), copper (CuNPs), and platinum (PtNPs), are among the most intensively studied in plant systems [29,65,66,67], largely due to their well-defined synthesis routes and pronounced physicochemical reactivity. AgNPs are valued for their strong antimicrobial properties, making them particularly effective in eliminating microbial contamination in in vitro cultures [23,68]. They also contribute to improved germination, shoot regeneration, and abiotic stress resistance by modulating redox signaling pathways [17,24,69].

Au NPs are widely applied in plant biotechnology owing to their high stability, biocompatibility, and tunable surface chemistry [25,26,70,71]. They enable efficient, non-viral delivery of genetic and regulatory molecules with reduced tissue damage and are also employed in biosensing and imaging applications through their distinctive plasmonic properties. CuNPs show promise as nanoagrochemicals, where low concentrations can enhance plant growth, nutrient uptake, and photosynthetic efficiency, while providing antimicrobial activity against phytopathogens, particularly when synthesized via green methods [27,30,72]. In contrast, PtNPs remain relatively underexplored in plant systems despite their high catalytic activity and potential utility in sensing and delivery; this highlights a gap in understanding PtNP–plant interactions and long-term physiological effects [28,29,73].

Metal oxide nanoparticles, such as zinc oxide (ZnO), titanium dioxide (TiO_2_), iron oxide (Fe_3_O_4_), cerium oxide (CeO_2_), aluminum oxide (Al_2_O_3_), and silicon dioxide (SiO_2_), offer chemical robustness, redox tunability, and wide-ranging functionality (Figure 2) [42,74]. Fe_3_O_4_ magnetic nanoparticles enable magnetic-field-assisted targeting and have been applied in magnetically guided delivery in plants such as pollen magnetofection [37,38,75,76]. In cryobiology more broadly, iron oxide NPs can be co-administered with cryoprotectant solutions to improve thermal control during rewarming (nanowarming), reducing devitrification-related injury; however, analogous magnetic targeting of cryoprotectant uptake in plant cryopreservation remains underexplored [77]. Al_2_O_3_ and SiO_2_ NPs support nutrient transport and cellular architecture, enhancing membrane integrity and shoot development in both in vitro and field contexts [40,41,78,79,80,81].

**Figure 3:**
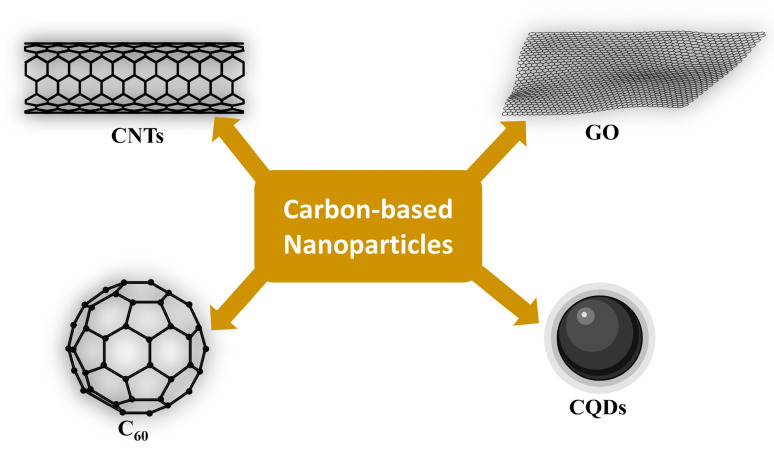
Classification of carbon-based nanoparticles commonly used in plant biotechnological systems and their structural representations. CNTs—Carbon Nanotubes; GO—Graphene Oxide; CQDs—Carbon Quantum Dots; C_60_—Fullerenes.

Carbon-based nanomaterials, including carbon nanotubes (CNTs), graphene oxide (GO), fullerenes, and carbon quantum dots (CQDs), are widely explored due to their high surface area, mechanical strength, and electronic conductivity (Figure 3) [44,45,46,82,83,84]. CNTs penetrate plant cell walls with minimal disruption and have been used for DNA and RNA delivery, often outperforming traditional biolistics. GO, reduced GO, and their composites offer controlled release properties and stimulate cell division in tissue cultures, though their oxidative reactivity requires concentration control. Fullerenes (e.g., C_60_) serve as potent reactive oxygen species (ROS) scavengers, stabilizing cells under stress. CQDs combine fluorescence with low toxicity, making them suitable for biosensing, tracking, and gene delivery in plant systems.

**Figure 4:**
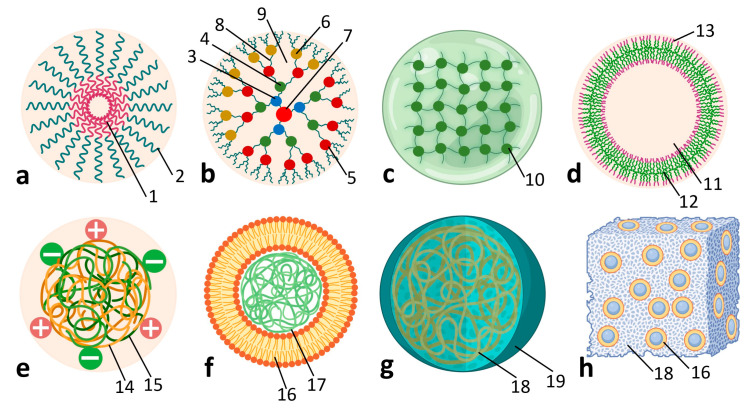
Polymeric nanoparticle functionalization strategies for targeted delivery. (**a**) Micelle; (**b**) Dendrimer; (**c**) Nanogel; (**d**) Polymersome; (**e**) Polyplex; (**f**) Lipopolyplex; (**g**) Nanosphere; (**h**) Cubosome; 1—hydrophobic block; 2—hydrophilic block; 3—first generation; 4—second generation; 5—third generation; 6—fourth generation; 7—core; 8—interior branch; 9—internal cavities; 10—crosslinked; 11—aqueous core; 12—hydrophobic membrane; 13—hydrophilic membrane; 14—cationic polymer; 15—anionic polymer; 16—lipid bilayer; 17—polymeric core; 18—hydrophilic polymer network; 19—polymer surface. Color coding is used to visually distinguish different material components and structural features and does not imply relative abundance, activity, or functional hierarchy.

Polymeric nanocarriers offer a highly adaptable and biodegradable platform for delivering genetic material, phytohormones, and cryoprotectants. As shown in Figure 4, advanced architectures such as micelles, dendrimers, nanogels, polymersomes, polyplexes, and cubosomes are used for targeted and controlled release, often in response to pH, temperature, or environmental stimuli [47,48,49,50,51,64,85,86,87].

Biogenic nanoparticles synthesized via green methods using plant extracts, fungi, algae, or bacteria offer eco-friendly, intrinsically bioactive alternatives. They can carry phytochemicals that enhance antioxidant defense, promote germination, or stimulate root elongation. However, their scalability and physicochemical consistency remain key hurdles for commercialization [52,53,54,55,56,57,58].

**Figure 5:**
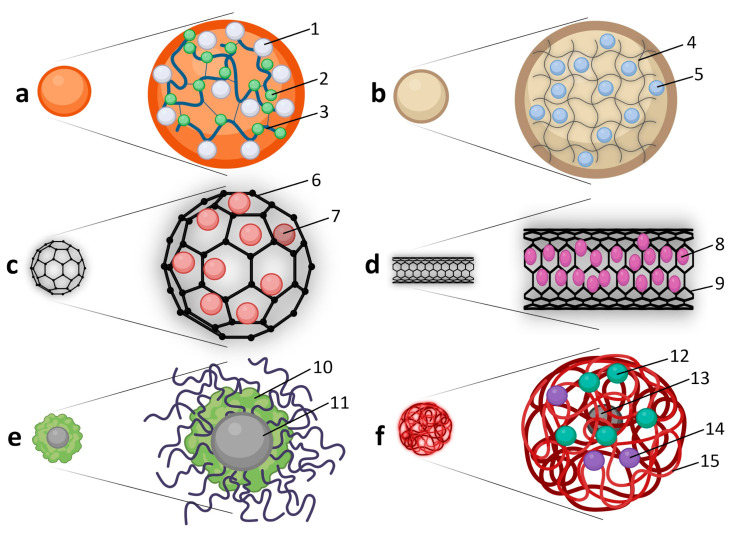
Representative hybrid and composite nanoparticle systems applied in plant science. (**a**) Silver–chitosan NP; (**b**) Zinc–alginate NP; (**c**) TiO_2_–graphene NP; (**d**) Fe_3_O_4_–CNT; (**e**) Core–shell structure NP; (**f**) Layered or matrix-embedded composites; 1—AgNPs; 2—NH_2_ groups; 3—Cross-linked chitosan; 4—Alginate; 5—Zn NPs; 6—Graphene oxide NP; 7—TiO_2_ NPs; 8—Fe_3_O_4_ NPs; 9—CNT; 10—PEG; 11—Magnetic Fe_3_O_4_ NPs complex; 12—Proline; 13—Metal NP; 14—Trehalose; 15—Polymer. Color coding is used to visually distinguish different material components and structural features and does not imply relative abundance, activity, or functional hierarchy.

Hybrid and composite nanoparticles integrate the functional advantages of multiple material classes to overcome limitations of single-component systems [59,60,61,62,63,64,88,89,90]. Examples include Ag–chitosan and Zn–alginate nanoparticles for antimicrobial action and micronutrient delivery, TiO_2_–GO and Fe_3_O_4_–CNT composites for synergistic photocatalytic or magnetic functionality, and core–shell or layered systems designed for improved stability and sequential release of cryoprotectants and antioxidants (Figure 5). These systems are particularly promising for complex applications such as cryopreservation, seed coating, and transformation, where multifunctionality and targeting are essential, although they also introduce additional challenges related to standardization, biocompatibility, and environmental degradation.

## 3. Nanoparticle Delivery Pathways, Uptake Mechanisms, and Localization in Plants

### 3.1. Delivery Methods of Nanoparticles in Plants

**Figure 6:**
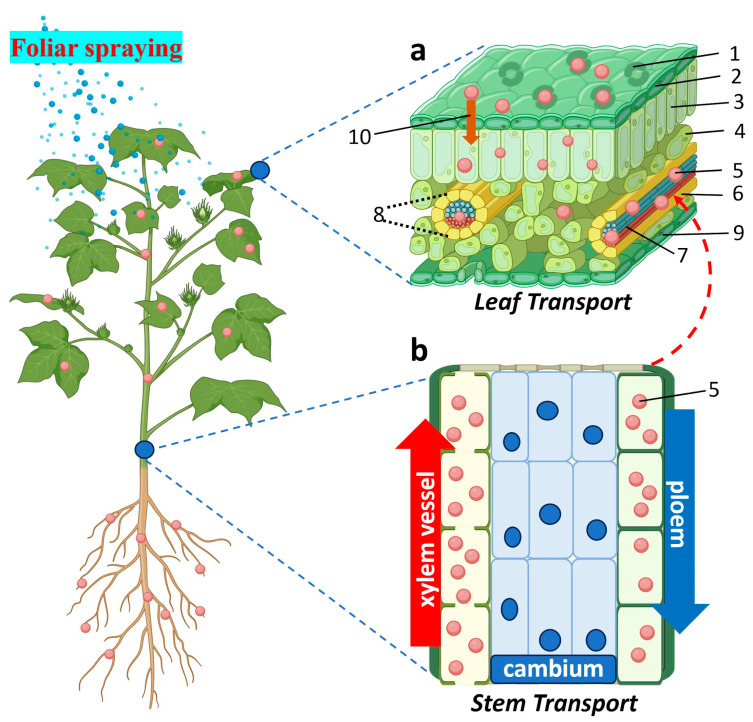
Schematic representation of the major pathways by which nanoparticles enter and move through aerial plant tissues following foliar (**a**) or stem (**b**) application. After deposition on the leaf surface, NPs may penetrate through cuticular imperfections or enter directly via stomatal openings (1), reaching the upper epidermis (2), palisade mesophyll (3), and spongy mesophyll (4). Once within the leaf apoplast, NPs (5) migrate through cell wall matrices and intercellular spaces, governed by size, surface charge, and aggregation state. Smaller or surface-functionalized NPs may cross the plasma membrane via endocytosis (10), enabling access to the symplast and subsequent cell-to-cell movement. Within the vascular bundle, NPs can be loaded into phloem (6) for bidirectional transport or into xylem vessels (7) for transpiration-driven upward movement. Redistribution through leaf veins (8) and toward the lower epidermis (9) supports systemic translocation to adjacent organs. The figure illustrates the structural barriers, entry routes, and vascular loading processes that collectively determine NP fate in aerial tissues, including apoplastic flow, symplastic transport via plasmodesmata, and long-distance movement through plant vasculature.

**Figure 7:**
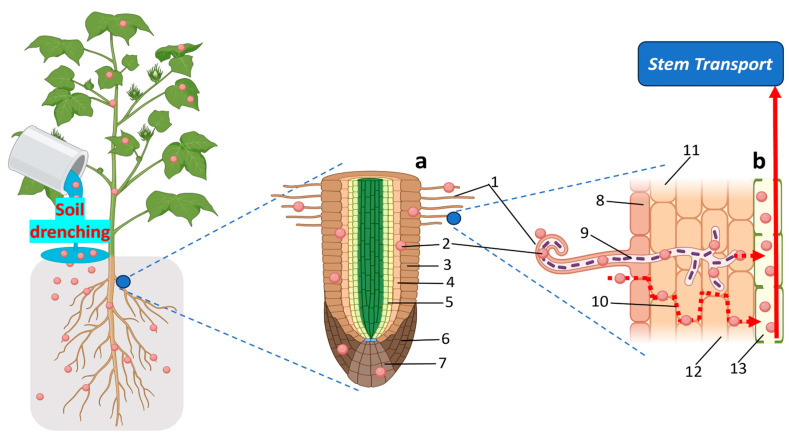
Schematic representation of nanoparticle (NP) absorption and transport through the root system. (**a**) Longitudinal section of a root illustrating NP entry from the rhizosphere and movement across major root tissues toward the stele, including root hairs (1), rhizosphere–root interface (2), epidermis (3), cortex (4), endodermis (5), lateral root cap (6), columellar root cap (7), and the vascular cylinder. (**b**) Enlarged view of NP transport at the cellular level, highlighting ectodermal cells (8), symplastic transport through plasmodesmata (9), apoplastic movement through cell walls and intercellular spaces (10), cortical cells (11), the endodermis and Casparian strip (12), and loading into the xylem (13) for upward, transpiration-driven stem transport.Following application to the rhizosphere, NPs interact with the root surface and enter root tissues via apoplastic or symplastic pathways, including membrane crossing and endocytosis, depending on NP size, charge, and surface functionalization. The endodermis acts as a selective barrier regulating access to the stele, where NPs or NP-derived species are translocated through the vascular system.

**Figure 8:**
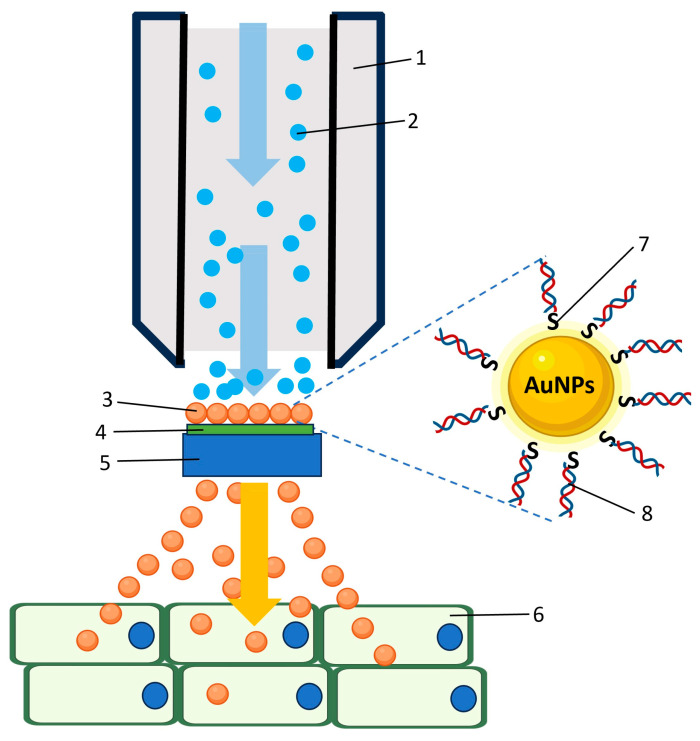
Schematic representation of the biolistic particle delivery system illustrating its working principle for introducing genetic material into plant cells. 1—gas acceleration tube; 2—helium; 3—AuNPs with DNA; 4—macrocarriers after the bombardment; 5—stopping screen; 6—target plant tissues; 7—thiol group; 8—DNA. Color coding is used for visual guidance to distinguish components and steps of the biolistic delivery system and does not imply quantitative relationships or relative importance.

**Figure 9:**
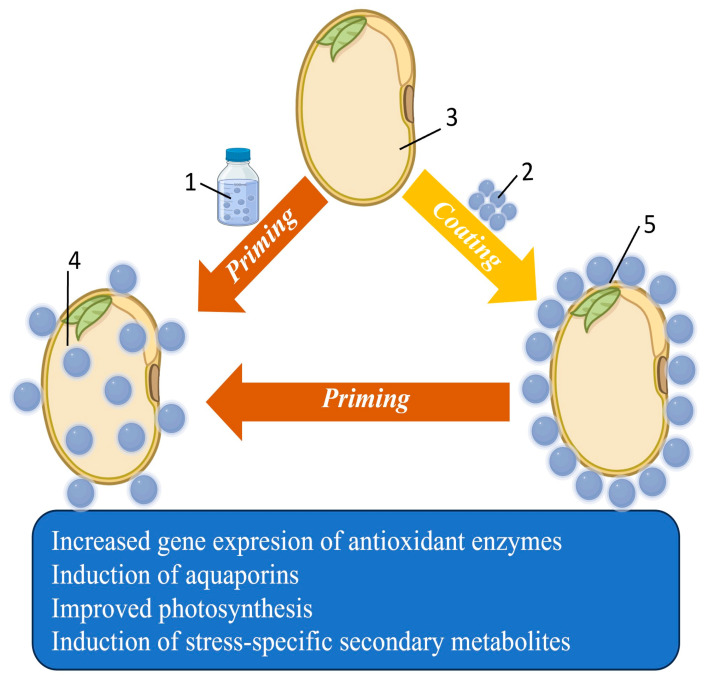
Schematic representation of seed nanopriming and nanocoating processes. 1—nanopriming solution; 2—dry powder of NPs; 3—seed; 4—seed nanopriming; 5—seed nanocoating. Color coding is used solely for visual grouping of application steps and materials and does not imply quantitative differences or relative importance.

Efficient delivery of NPs into plant systems is pivotal for realizing their functional potential in biotechnology, crop enhancement, and cryopreservation. A wide range of physical, chemical, and biological methods has been developed to facilitate this process, with the choice of delivery strategy often tailored to the plant species, developmental stage, tissue type, and intended application [91,92]. Figure 6, Figure 7, Figure 8 and Figure 9 illustrate the principal routes of NP entry, internal transport, and application platforms.

Foliar spraying is a widely used, non-invasive method of delivering NPs directly to the aerial parts of plants. This method is especially effective for rapid uptake of bioactive NPs involved in micronutrient supplementation, disease control, stress modulation, and even gene delivery (Figure 6) [91,92]. Following deposition on the leaf surface, NPs may enter through stomata, cuticular imperfections, or trichomes and subsequently migrate through epidermal and mesophyll tissues before reaching vascular elements. Cellular internalization can occur via endocytosis, enabling active translocation and, in some cases, systemic distribution throughout the plant.

Uptake efficiency following foliar application is governed by particle size, surface charge, and hydrophilicity, as well as environmental factors such as humidity, leaf morphology, and cuticle properties. Despite its versatility, foliar delivery presents challenges including non-uniform deposition, droplet runoff, photodegradation, and potential phytotoxicity [93,94]. Accordingly, formulation optimization, such as the use of surfactants, stabilizers, and controlled-release systems, is critical for improving efficacy and minimizing unintended effects.

Root-based NP delivery leverages the plant’s natural water and nutrient absorption mechanisms, providing a sustained, systemic delivery pathway through soil or hydroponic systems (Figure 7) [91,95]. In the rhizosphere, NPs interact with root hairs and epidermal cells and can move through apoplastic and symplastic pathways before entering the vascular tissue for upward translocation via the xylem. NP behavior in this environment is strongly influenced by particle properties and interactions with soil microbes, root exudates, and organic matter, which may alter NP stability, aggregation, or dissolution [96,97,98]. Because it closely mimics environmental exposure scenarios, root application is particularly relevant for nanofertilizers, growth stimulants, and abiotic stress mitigation strategies.

Microinjection provides direct cytoplasmic delivery of NP suspensions using fine micropipettes, bypassing external barriers such as cell walls and cuticles [14,99,100]. Although technically demanding and limited in throughput, this approach allows precise targeting of meristematic tissues, embryos, or zygotes and is valuable for mechanistic studies, gene delivery, and localized cryoprotectant introduction. Vacuum- or pressure-assisted infiltration methods offer a less invasive alternative by facilitating NP diffusion into intercellular spaces, particularly in leaves or callus tissues. These approaches support the delivery of functionalized NPs carrying DNA, RNA, proteins, or siRNAs and are increasingly used in *Agrobacterium*-free expression platforms, although their application remains largely experimental due to equipment and dosing constraints.

Biolistic delivery, or particle bombardment, is a powerful physical method for introducing NPs into plant cells using high-velocity propulsion of coated micro- or NPs [101,102]. In this system, helium-driven acceleration propels functionalized gold nanoparticles toward target tissues, enabling direct intracellular delivery (Figure 8). Biolistics is particularly useful for recalcitrant species, including many monocots, and supports DNA-free CRISPR/Cas delivery, protein transfer, and nanocarrier evaluation [103,104,105,106,107]. However, tissue damage, equipment cost, and limited targeting precision remain important limitations, requiring careful optimization of bombardment parameters.

Seed-based delivery platforms, nanopriming, and nanocoating enable pre-sowing incorporation of NPs, representing cost-effective and scalable approaches for early-stage interventions [108,109,110]. Seed priming involves soaking seeds in NP-containing solutions to initiate metabolic activation, whereas nanocoating applies NP formulations within polymer matrices to enable gradual release during germination (Figure 9). These methods have been shown to enhance germination, seedling vigor, and stress tolerance, and may also influence rhizosphere interactions. For example, ZnO and Fe_3_O_4_ nanoparticles can support enzymatic activity and chlorophyll synthesis, carbon-based nanomaterials may improve water uptake and nutrient mobilization, and chitosan nanoparticles provide antimicrobial protection and stimulate defense-related gene expression.

Despite their promise, seed-based and other NP delivery approaches raise concerns regarding dose uniformity, persistence, environmental fate, and long-term biosafety. As regulatory frameworks evolve, rigorous safety evaluation and formulation standardization will be essential for responsible deployment.

In summary, NP delivery methods in plant systems range from scalable foliar and root-based applications to highly precise but low-throughput experimental techniques such as microinjection. Selection of an appropriate delivery strategy must balance biological effectiveness, scalability, and environmental compatibility. Continued advances in formulation design, tissue targeting, and eco-safety assessment will be critical for translating these approaches from experimental tools into practical solutions for sustainable plant biotechnology.

### 3.2. Uptake and Localization in Plants

Once NPs enter the plant system, whether through foliar application, root absorption, seed treatment, infiltration, or microinjection, their subsequent movement and distribution determine their biological activity, effectiveness, and safety. Understanding how NPs traverse plant tissues, reach target cells, and accumulate within specific organelles is essential for optimizing their design for applications such as gene delivery, nutrient supplementation, regeneration enhancement, or cryoprotection. Their fate in planta is strongly influenced by physicochemical properties (size, shape, charge, surface coating) as well as plant-related factors including species, tissue type, developmental stage, and surrounding environmental conditions [91,111].

NP behavior within plants typically proceeds through several stages: cellular uptake, intracellular trafficking, long-distance vascular transport, and final localization in specific compartments. Together, these processes determine whether NPs successfully reach intended targets such as meristematic tissues, chloroplasts, or the vascular system.

Nanoparticles can enter plant cells through multiple routes, each shaped by the interplay between cell wall architecture and NP properties [91,111]. Apoplastic entry occurs when NPs move through the cell wall and intercellular spaces without crossing the plasma membrane. This route supports the passive diffusion of small particles that can navigate the porous cellulose–lignin network. Because the cell wall typically restricts particles larger than 5–20 nm, apoplastic movement is most efficient for small, dispersed NPs. Although confined to the cell wall region, such NPs may still influence signaling or cell wall-associated processes.

Symplastic entry involves passage from cell to cell through plasmodesmata, requiring prior crossing of the plasma membrane. While plasmodesmatal channels are highly size-restricted (~2–3 nm), their conductance can fluctuate depending on developmental state or stress [112,113]. Consequently, most engineered NPs require membrane translocation before accessing this pathway.

Endocytosis represents a central active mechanism for NP internalization, particularly for small (<50 nm), surface-modified, or positively charged particles. These NPs interact with membrane components to trigger vesicle formation, allowing uptake via clathrin-mediated or caveolae-like pathways [114,115]. Once internalized, vesicles can traffic to various organelles or fuse with vacuoles, influencing NP stability and activity. Mechanisms such as lipid exchange envelope penetration also explain entry into double-membrane organelles under certain surface-charge conditions [116].

Uptake efficiency is shaped by NP features such as small size, spherical morphology, mild positive charge, and plant-compatible surface functionalization. Ligand or peptide coatings further enhance specificity and internalization by facilitating interactions with plant membrane receptors [114,117].

After entering tissues, NPs may move long distances through the plant’s vascular system. This systemic mobility is crucial for applications requiring whole-plant delivery, such as nutrient distribution or plant-wide stress protection.

The xylem serves as the primary route for upward transport from roots to shoots, driven by transpiration. Hydrophilic and negatively charged NPs, including many metal oxides, readily enter this pathway and travel with the transpiration stream [118]. Xylem mobility has been demonstrated in several species, enabling redistribution to stems, leaves, and aerial tissues.

The phloem enables bidirectional transport between source and sink tissues and may carry smaller, neutrally charged, or specially functionalized NPs. Surface modifications, such as PEGylation, improve NP stability in phloem sap and reduce aggregation, thereby facilitating movement and delivery to meristematic or storage tissues [114].

Beyond physical barriers and bulk flow in xylem and phloem, molecular transport systems can influence nanoparticle uptake from soil and their delivery to sink organs, including grains, especially when nanomaterials partially dissolve or transform in the rhizosphere [91,95,96,97,98,111]. For many metal and metal oxide formulations used as nanofertilizers, a substantial fraction of the material can contribute to the ionic pool through dissolution, complexation with root exudates, or surface transformation, and the resulting ions are taken up by canonical nutrient transporters in root epidermal and cortical cells [91,95,111]. In these cases, the plant does not transport intact nanoparticles exclusively, but rather a mixture of particulate forms and nanoparticle-derived ions, and the contribution of each fraction depends on particle size, coating chemistry, aggregation state, and soil conditions [95,111].

Transporter involvement is therefore most strongly supported for the nanoparticle-derived ionic fraction, because micronutrient uptake is mediated by established membrane transport families [91,92,95,111]. For example, zinc, iron, and manganese released from ZnO, Fe-based, or Mn-based nanomaterials can enter root cells through metal uptake systems that normally operate in mineral nutrition, and these same transporter networks contribute to subsequent redistribution within the plant via xylem loading and remobilization to sinks [91,95,111]. In parallel, water transport capacity and membrane permeability, which are regulated in part by aquaporins, can modulate the movement of small solutes and influence overall uptake dynamics in roots, and carbon-based nanomaterials have been associated with altered expression of water channel genes in exposed plants. These observations support the view that nanomaterials can affect uptake not only through direct penetration or endocytosis, but also through indirect regulation of membrane transport physiology [91,92].

For intact nanoparticles that remain largely particulate, current evidence indicates that cellular entry is dominated by apoplastic movement to the endodermis followed by membrane crossing via endocytosis or other active internalization routes, rather than by passage through classical ion transporters that are selective for small solutes [91,111,114,115]. Once NPs or their transformed products reach the stele, upward transport is typically driven by xylem flow, while delivery to strong sinks is more consistent with phloem-mediated redistribution, which has been demonstrated for selected nanomaterials under controlled conditions [118]. Accumulation in reproductive tissues and developing grains therefore depends on the balance between xylem delivery, phloem exchange, sequestration in cell walls or vacuoles, and remobilization of any dissolved fraction, processes that are central to food safety assessment [94,111,118].

Final NP localization within the plant determines both functional efficacy and potential toxicity. Plant tissues commonly accumulate NPs in cell walls and vacuoles, which act as major sinks, especially for larger or unmodified particles. Such sequestration may limit bioactivity but also contributes to detoxification. Targeted delivery to specific organelles is possible when NPs are engineered with appropriate size or surface ligands. For instance, carbon nanotube-based systems have been used to reach chloroplasts, improving photosynthetic function or enabling plastid-targeted gene delivery [119]. Nuclear localization has also been achieved using NPs carrying nucleic acids or proteins functionalized with atomic localization sequences [117].

Localization is typically assessed using fluorescent labeling, electron microscopy, or elemental analysis techniques, each of which presents analytical challenges in differentiating internalized NPs from those adhering to surfaces [111]. While precise localization can enhance efficiency, unintended accumulation, particularly of persistent inorganic nanomaterials, can disrupt cellular homeostasis or induce oxidative stress. Therefore, careful control of NP size, charge, and functionalization is essential to minimize phytotoxicity while maximizing targeted delivery.

In summary, NP uptake, transport, and localization are key determinants of both functional performance and biological safety in plant systems. By tailoring nanoparticle properties to achieve precise delivery while ensuring controlled accumulation and clearance, the benefits of plant nanotechnology can be maximized while minimizing unintended consequences.

## 4. Nanoparticles in Plant Biotechnology

This section explores the expanding applications of NPs in plant biotechnology, focusing on four core areas: genetic engineering and transformation, plant tissue culture and regeneration, cryopreservation, and abiotic stress mitigation. These domains represent critical intersections where nanotechnology can significantly enhance the efficiency, precision, and sustainability of plant improvement strategies.

### 4.1. Nanoparticles in Plant Genetic Engineering and Transformation

Recent years have seen rapid progress in the application of NPs as carriers for genetic material in plants, enabling *Agrobacterium*-independent and often DNA-free transformation strategies. These approaches vary widely with respect to NP type, cargo, delivery method, and plant species, resulting in diverse transformation outcomes. Table 2 provides a comparative overview of representative NP-mediated transformation studies in plants, detailing the nanoparticle systems used, delivered cargos, application methods, concentrations, and key experimental outcomes. Nanotechnology provides a versatile alternative: it enables the transport of DNA, RNA, proteins, and CRISPR components into intact tissues with minimal injury and without relying on bacterial vectors [103,105,106,107]. Because nanoparticle properties can be precisely tuned through size control, surface chemistry, and functionalization, they provide an unprecedented level of control over cargo loading, cellular entry, and intracellular fate. Their emergence marks a significant shift in the conceptual framework of plant transformation.

One of the most transformative roles of NPs in plant biotechnology is their use as carriers for genetic material, offering a non-viral, non-biological alternative to conventional transformation systems. Traditional gene transfer approaches such as *Agrobacterium tumefaciens*-mediated transformation or biolistic delivery face notable constraints, including limited host range, risk of tissue damage, and reliance on marker genes [132].

Nanoparticles circumvent many of these limitations by enabling direct delivery of DNA, RNA, or proteins into plant cells with minimal collateral damage. Their small size (<100 nm), surface-modifiability, and compatibility with biomolecules make them ideal gene carriers across diverse plant species [117,133].

Magnetic NPs have been used for magnetofection, where external magnetic fields guide DNA-functionalized Fe_3_O_4_ particles into cells. This strategy supports transient and stable expression with reduced tissue trauma compared to biolistics [134].

Carbon nanotubes represent another important platform. Their high aspect ratio enables penetration through the cell wall, and their inner cavity allows encapsulation of nucleic acids. CNT-mediated delivery has enabled direct chloroplast and nuclear transformation without tissue culture, representing a breakthrough in in planta gene transfer [114].

Gold NPs are widely studied because of their inertness and biocompatibility [103,105,135]. They can bind nucleic acids and be delivered via foliar sprays, vacuum infiltration, or low-pressure biolistics. Au NPs provide multiplexing potential, allowing simultaneous delivery of multiple constructs.

CRISPR/Cas enables targeted and efficient genome modification, but conventional delivery methods suffer from low efficiency, tissue damage, and frequent integration of foreign DNA [136].

Nanoparticles provide species-independent, transient carriers for Cas9 proteins, single guide RNA (sgRNAs), or ribonucleoprotein (RNP) complexes. Carbon dots have delivered CRISPR/Cas9 RNPs into protoplasts with successful edits and minimal off-target effects [117]. Mesoporous silica NPs offer large cargo capacity for plasmid DNA and RNPs with controlled release [129,137]. Lipid-based nanocarriers, long used in mammalian systems, are being adapted for plants. PEGylated AuNPs stabilize and protect Cas9/gRNA, promoting nuclear import and transient activity [117].

A key advantage is DNA-free editing, avoiding transgene integration and enhancing regulatory acceptance. Nanoparticle carriers also enable spatial and temporal control of editing by targeting specific tissues or developmental stages [117,138].

In summary, NP-mediated gene delivery offers a precise, marker-free, and species-flexible platform with broad potential for plant transformation.

### 4.2. Nanoparticles in Plant Tissue Culture and Regeneration

Nanoparticles are emerging as potent modulators of plant morphogenesis in vitro, capable of reshaping fundamental processes that govern callus formation, somatic embryogenesis, and shoot regeneration [78,79,139,140]. Their influence arises from a combination of physicochemical interactions with plant cells, modulation of intracellular signaling pathways, and the ability to deliver bioactive compounds directly into developing tissues. As a result, nanotechnology is rapidly moving beyond its role as a delivery tool and becoming a strategic component in modern tissue culture systems aimed at improving regeneration efficiency, reducing recalcitrance, and stabilizing developmental pathways under in vitro stress.

The integration of carbon-based nanomaterials into regeneration systems has offered some of the most compelling evidence for NP-mediated enhancement of morphogenesis. Carbon nanotubes, in particular, have demonstrated high compatibility with plant cells and tissues. Early studies showed their ability to penetrate cell walls, localize within the cytoplasm and organelles, and alter cellular metabolism without inducing catastrophic toxicity [122]. Building on this foundation, Khodakovskaya and colleagues revealed that CNT exposure can stimulate cell division, reprogram metabolic activity, and modify the expression of genes associated with growth and stress response [141,142]. These effects are not superficial; instead, they influence key parameters that directly determine tissue culture success, including water uptake, hormonal responsiveness, and maintenance of cellular totipotency. CNT-induced modulation of aquaporin expression in tomato plants [143] further highlights the capacity of carbon NPs to reconfigure cellular transport processes to promote regeneration.

Studies with plant suspension cells have provided parallel insights. Work on Arabidopsis and rice cell cultures has shown that multi-walled CNTs, even at moderate concentrations, are readily internalized and can alter intracellular redox status, mitochondrial activity, and metabolic fluxes. These factors determine whether cells transition toward callus proliferation or remain quiescent [125,126]. These findings suggest that carbon-based NPs act at a regulatory level, influencing early morphogenic signals rather than merely serving as passive carriers of biomolecules.

Fullerene NPs add a distinct dimension to this field because of their pronounced antioxidant properties. Their biphasic effects on ROS scavenging, chlorophyll fluorescence, and water relations in maize mirror critical physiological challenges encountered by explants during sterilization, wounding, or initial culture establishment [144]. Since oxidative stress is a significant cause of cell death and regeneration failure, fullerene NPs and other ROS-modulating nanomaterials offer a targeted strategy. They can protect vulnerable tissues, stabilize embryogenic cells, and improve overall regeneration competence. Their application parallels advance in cryopreservation, where NPs have been used to mitigate ROS accumulation and maintain post-thaw viability, underscoring the shared biochemical basis of tissue survival in both contexts.

Metal oxide NPs, such as ZnO, TiO_2_, and CeO_2_, have also been shown to influence key developmental pathways in vitro [145,146,147,148]. Their interactions with auxin transport, antioxidant enzyme activity, and nutrient homeostasis position them as potent regulators of callus behavior and shoot formation. Low concentrations frequently stimulate organogenesis by maintaining optimal ROS levels and improving cellular energy metabolism. In contrast, excessive doses can induce stress or cytotoxicity, revealing a narrow yet highly effective operational window. Their multifunctionality makes them valuable tools for fine-tuning regeneration protocols in recalcitrant genotypes.

Despite these promising advances, the application of NPs in regeneration still faces critical challenges. Optimal concentrations vary widely among species, and dose–response relationships are often non-linear. Some NPs may persist in regenerants longer than expected, raising questions about their long-term physiological effects. Moreover, the variability in NP synthesis methods complicates reproducibility across laboratories. Nonetheless, the consistent trends observed across multiple studies indicate that, when precisely controlled, NPs can significantly enhance the responsiveness and stability of tissue culture systems.

As plant biotechnology shifts toward increasingly efficient transformation and regeneration pipelines, nanoparticles provide a robust set of tools to address long-standing limitations in species recalcitrance, explant stress responses, and regeneration fidelity. Their ability to modulate fundamental developmental signals in vitro positions them not merely as additives, but as strategic components of next-generation regeneration systems capable of supporting both basic research and large-scale plant improvement programs.

### 4.3. Nanoparticles in Plant Cryopreservation

Cryopreservation underpins modern plant biotechnology and germplasm conservation, offering a reliable means to preserve elite cultivars, endangered species, and recalcitrant tissues at ultra-low temperatures without genetic drift or physiological deterioration. Yet the full potential of cryopreservation is constrained by the intrinsic vulnerability of plant cells to freezing and thawing stresses. Cryoinjury arises from a convergence of damaging events, including intracellular ice formation, osmotic imbalance, membrane phase transitions, and pronounced oxidative stress. Although conventional cryoprotectants mitigate some of these effects, they exhibit inherent toxicity at high concentrations and often fail to safeguard sensitive, meristematic, or embryogenic tissues [9,149]. This persistent limitation has prompted the search for more sophisticated, multifunctional cryoprotective agents. The integration of NPs into plant cryopreservation protocols has emerged as a promising strategy to mitigate cryoinjury, particularly oxidative stress, membrane damage, and post-thaw metabolic imbalance [150,151]. Although still limited in scope, published studies demonstrate that different NP types can significantly improve survival and regeneration after liquid nitrogen (LN) exposure. Table 3 summarizes reported applications of NPs in plant cryopreservation, including the plant material treated, NP type and concentration, cryopreservation method, and the main physiological and biochemical outcomes. Their nanoscale dimensions, extraordinary surface reactivity, and tunable physicochemical properties enable interactions with plant cells at levels of precision unattainable with traditional cryoprotectants. Rather than acting as passive additives, NPs intervene directly in the biophysical and biochemical processes that determine cryopreservation success, offering a comprehensive protective strategy that spans antioxidant defense, membrane stabilization, osmotic buffering, thermal regulation, and post-thaw metabolic recovery.

Oxidative stress represents one of the most severe and universal forms of cryodamage. During exposure to vitrification solutions, ultra-rapid cooling, and thawing, cellular redox balance collapses and triggers massive bursts of ROS, including superoxide, hydrogen peroxide, and hydroxyl radicals. These species drive lipid peroxidation, protein denaturation, DNA fragmentation, and membrane rupture. As Ren et al. emphasized, the magnitude of ROS accumulation is often the decisive factor determining whether tissues survive cryostorage [150]. The functional relevance of these mechanisms has been demonstrated in plant systems. In Agapanthus praecox, both carbon NPs and single-walled carbon nanotubes substantially increased post-LN survival by reducing ROS and limiting membrane leakage [150,152]. Fullerene-based NPs similarly reduced oxidative damage and improved tissue stability in stressed maize, underscoring the cross-species utility of nanomaterials as redox regulators.

Beyond direct cellular effects, nanoparticle–cryoprotectant interactions represent a critical and often underappreciated determinant of cryopreservation outcomes. Nanoparticles can influence the physicochemical properties of vitrification solutions, including viscosity, thermal conductivity, ice nucleation behavior, and heat transfer during cooling and rewarming. In cryobiology more broadly, iron oxide nanoparticles have been exploited for nanowarming to reduce devitrification-related injury during rewarming; however, the systematic application of such thermal control strategies in plant cryopreservation remains largely unexplored [77]. These observations highlight that nanoparticle-assisted cryoprotection arises from coupled biophysical and biochemical effects rather than from oxidative stress suppression alone.

Despite encouraging proof-of-concept results, nanoparticle-assisted plant cryopreservation remains at an early developmental stage. Key challenges include variability in nanoparticle synthesis and characterization, incomplete understanding of NP persistence or removal after thawing, potential interactions with vitrification solution components, and limited cross-species reproducibility. Moreover, most studies have been conducted under highly controlled experimental conditions, and the scalability of NP-assisted cryopreservation protocols has yet to be demonstrated.

In summary, nanoparticles offer a versatile and mechanistically diverse toolkit for improving plant cryopreservation outcomes by modulating membrane stability, osmotic balance, redox homeostasis, and thermal behavior during freezing and rewarming. However, their successful integration into routine cryobanking will require systematic comparison of nanoparticle classes, standardized reporting of physicochemical properties, and rigorous evaluation of long-term biological safety and reproducibility across laboratories.

### 4.4. Nanofertilizers and Stress Mitigation

While redox regulation and membrane protection are especially critical during cryopreservation, these same mechanisms also contribute to nanoparticle-mediated tolerance to drought, salinity, and nutrient stress under non-cryogenic conditions. In this context, nanoparticles have emerged as effective tools for enhancing plant stress resilience and improving nutrient-use efficiency, leading to the development of materials commonly described as nanofertilizers and nanostimulators [144,154,155]. Their unique physicochemical features, including high surface area, increased solubility, and the ability to interact intimately with plant cellular structures, enable precise modulation of physiological processes that conventional fertilizers or agrochemicals cannot achieve. Rather than functioning solely as nutrient sources, NPs actively reshape cellular metabolism, redox balance, and signaling networks, thereby strengthening plant tolerance to drought, salinity, heavy metals, temperature extremes, and oxidative stress.

A major pathway through which nanoparticles mitigate stress is by regulating ROS and enhancing antioxidant defense systems [22,151,156,157,158,159]. Metal oxide NPs such as ZnO, TiO_2_, CeO_2_, and Fe_3_O_4_ possess inherent redox-buffering capacities. Cerium oxide NPs are particularly notable for their reversible Ce^3+^/Ce^4+^ cycling, which allows continuous ROS scavenging in a self-regenerating manner. By suppressing excessive ROS accumulation during abiotic stress, these nanoparticles help maintain membrane stability, preserve chloroplast function, and support cell viability. Such biochemical stabilization leads to improved photosynthetic rates, enhanced growth, and greater survival in both in vitro cultures and whole plants.

In addition to direct ROS buffering by redox-active nanomaterials, stress protection by nanoparticles involves coordinated activation of plant molecular defense networks that regulate antioxidant capacity, osmotic adjustment, ion homeostasis, and stress-responsive gene expression [22,156,157,158,159]. At the antioxidant level, nanoparticle exposure has been associated with increased activity and transcriptional induction of enzymatic scavengers, including superoxide dismutase, catalase, ascorbate peroxidase, glutathione peroxidase, peroxidases, and glutathione reductase, which collectively sustain the ascorbate glutathione cycle and limit lipid peroxidation under drought, salinity, heat, and metal stress [156,157,158,159]. These responses reduce membrane-damage markers, such as malondialdehyde, stabilize chloroplast ultrastructure, and preserve photosystem performance, consistent with the improved photosynthetic rates reported for multiple nanoformulations used as nanofertilizers or nanostimulators [22,157,158,159]. Importantly, the direction and magnitude of these effects depend on nanoparticle dose and physicochemical properties, and low-dose exposures can act as priming stimuli that accelerate subsequent defense activation during stress, consistent with hormetic dose–response behavior reported for diverse nanomaterials in plants.

Nanoparticles also support molecular acclimation by influencing osmotic and water balance mechanisms that are central to drought and salinity tolerance [156,157,158,159]. Studies compiled in recent reviews indicate that nanofertilizers can increase the accumulation of compatible solutes such as proline and soluble sugars, improve relative water content, and modulate stomatal regulation, thereby sustaining turgor and limiting dehydration-induced oxidative cascades [157,158]. Carbon-based nanomaterials have additionally been linked with changes in aquaporin associated water transport and improved hydration status under drought conditions, which supports a membrane transport component to NP-mediated tolerance [22].

Under salinity and metal stress, protection often involves maintaining nutrient and ion balance, which reduces secondary oxidative damage [156,157,158,159]. Nanofertilizer-derived micronutrients can restore cofactor availability for antioxidant enzymes and photosynthetic machinery, while silicon and iron-based nanoformulations have been associated with improved ionic homeostasis and reduced uptake or internal toxicity of harmful ions in stressed plants [22,156,157,158,159]. At the signaling level, nanoparticles can interact with phytohormone and stress signaling networks, including abscisic acid-linked pathways that regulate stomatal behavior, root system architecture, and transcriptional activation of stress-responsive genes [22,156,157,158]. Together, these molecular responses explain how nanoparticles function not only as nutrient sources, but also as stress response modulators that integrate redox homeostasis, osmoprotection, transport physiology, and gene regulation to enhance abiotic stress resilience [22,156,157,158,159].

**Figure 10:**
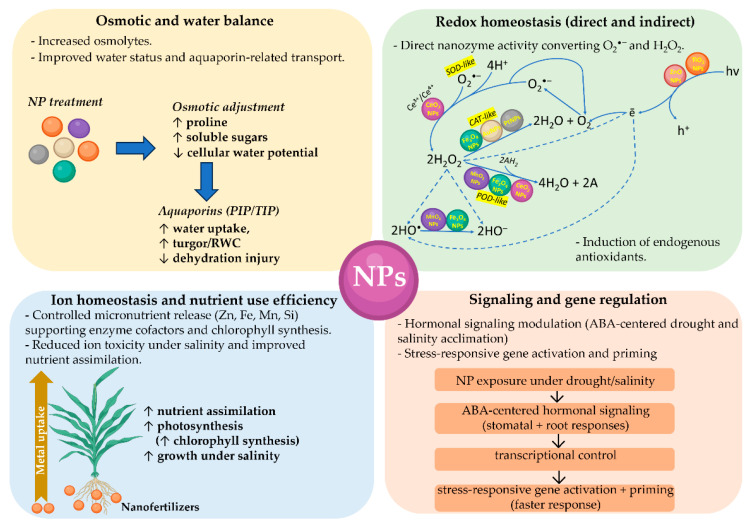
Conceptual overview of major mechanisms through which nanoparticles (NPs) can enhance plant tolerance to abiotic stress. The figure summarizes representative and non-exclusive pathways, including osmotic adjustment and water balance, redox homeostasis via direct nanozyme activity and induction of endogenous antioxidants, ion homeostasis and nutrient use efficiency, and hormonal signaling–mediated gene regulation and priming. The relevance and contribution of each mechanism depend on nanoparticle composition, dose, formulation, plant species, and stress context. ABA, abscisic acid; PIP, plasma membrane intrinsic protein (aquaporin subfamily); TIP, tonoplast intrinsic protein (aquaporin subfamily); RWC, relative water content; O_2_^•−^, superoxide radical; H_2_O_2_, hydrogen peroxide; HO^•^, hydroxyl radical; HO^−^, hydroxide ion; AH_2_/A, reduced/oxidized antioxidant substrate pair (generic notation used in peroxidase-type reactions). Arrows indicate representative interactions among pathways, while color coding and bold text are used for visual grouping and do not imply quantitative weighting.

Nanoparticles mitigate abiotic stress in plants through a coordinated set of physiological, biochemical, and molecular mechanisms rather than through antioxidant activity alone. As summarized in Figure 10, NP-mediated stress tolerance integrates regulation of redox homeostasis, osmotic and water balance, ion homeostasis, nutrient-use efficiency, and stress-responsive signaling pathways. NPs can act both directly, through enzyme-like catalytic activities that modulate ROS, and indirectly by priming endogenous defense systems, hormonal signaling, and transcriptional responses. At the redox level, several metal and metal oxide NPs exhibit nanozyme-like properties, functioning as mimics of antioxidant enzymes such as superoxide dismutase, catalase, and peroxidases. These activities contribute to controlled ROS detoxification while maintaining signaling-competent redox states. In parallel, NP exposure induces endogenous antioxidant systems, reinforcing cellular redox buffering capacity. Beyond redox regulation, NPs promote osmotic adjustment by enhancing the accumulation of compatible solutes, improving cellular water potential and aquaporin-mediated water transport, which together support turgor maintenance and reduce dehydration injury under drought and salinity stress. Nanoparticles also influence ion homeostasis and nutrient-use efficiency by enabling controlled micronutrient delivery, supporting chlorophyll synthesis, enzymatic function, and photosynthetic performance while limiting ion toxicity under saline conditions. At the signaling level, NP exposure modulates stress-related hormonal pathways, particularly abscisic acid (ABA)-centered signaling, leading to coordinated stomatal and root responses. These upstream signals converge on transcriptional control and stress-responsive gene activation, resulting in physiological priming and faster acclimation to subsequent stress episodes. Together, the mechanisms illustrated in Figure 10 highlight NPs as multifunctional stress modulators that integrate redox balance, water relations, nutrient dynamics, and gene regulation to enhance plant resilience under drought and salinity conditions, rather than acting solely as passive antioxidants.

Nanoparticles also bolster stress tolerance by improving nutrient uptake, transport, and assimilation, processes often compromised under adverse environmental conditions [22,151,156,157,158,159]. As nanofertilizers, they supply essential micronutrients such as zinc, iron, manganese, and silicon in highly bioavailable forms. Their nanoscale size enables close interaction with root surfaces, penetration into root tissues, and controlled nutrient release, resulting in higher uptake efficiency and reduced nutrient losses. Improved nutrient availability supports chlorophyll synthesis, enzymatic activity, osmotic balance, and stomatal regulation, often at doses far lower than those required for conventional fertilizers, thereby minimizing toxicity and environmental contamination. Beyond nutrient delivery, NPs modulate plant hormonal and signaling networks. Metal oxide NPs such as ZnO and TiO_2_ influence auxin- and abscisic acid–mediated pathways that regulate root architecture, stomatal behavior, and stress acclimation. Carbon-based nanomaterials interact with membranes and aquaporins, enhancing water transport and cellular hydration during drought stress. Together, these effects contribute to improved physiological resilience under abiotic stress.

These functions have particularly significant implications for plant tissue culture, where explants are highly susceptible to oxidative shock, osmotic stress, and nutrient imbalance during sterilization and early in vitro development. Nanoparticles that buffer ROS, stabilize membranes, or supply micronutrients in controlled forms can markedly improve explant survival, callus quality, and regeneration efficiency. Fullerene-based NPs, for example, alleviate oxidative pressure in stressed maize tissues [144], while metal oxide nanofertilizers incorporated into culture media have been associated with enhanced embryogenic competence and more robust plantlet formation across multiple species.

Complementing their intrinsic bioactivity, nanofertilizers are increasingly engineered as smart, stimuli-responsive systems that release nutrients in response to environmental cues such as soil pH, temperature, or moisture levels. Biodegradable polymer coatings enable controlled swelling or degradation under specific conditions, ensuring nutrient delivery is synchronized with plant demand [160]. Such precision is equally advantageous in tissue culture, where the timing of nutrient availability can influence developmental transitions such as callus induction, somatic embryogenesis, and shoot regeneration.

The multifunctionality of nanofertilizers extends further when NPs are co-formulated with hormones, beneficial microbes, vitamins, or stress-protective molecules, creating integrated delivery platforms that simultaneously stimulate growth, enhance nutrient acquisition, and mitigate stress. These synergistic formulations hold particular promise for optimizing morphogenic responses in recalcitrant plant species.

Despite their considerable potential, the broader adoption of nanofertilizers requires careful attention to dosage, formulation, and species-specific responses [155,161,162,163]. Many nanoparticles exhibit biphasic effects, stimulating growth at low concentrations but causing toxicity or oxidative damage at higher levels. Their interactions with soil matrices, root exudates, or components of culture media can alter stability and uptake pathways. Concerns regarding nanoparticle accumulation in edible tissues and long-term effects on soil microbiota necessitate continued evaluation.

Overall, NP-based nutrient and stress-mitigation systems represent a significant technological advancement in plant science. By integrating precise nutrient delivery with redox regulation, hormonal modulation, and metabolic stabilization, nanofertilizers offer robust solutions for enhancing plant performance in both controlled in vitro environments and challenging field conditions. As synthesis and functionalization techniques continue to evolve, these nanomaterials have the potential to contribute to next-generation sustainable plant production and stress-resilient agriculture.

Across all application domains discussed in this section, nanoparticle-enabled effects should be interpreted as context-specific and formulation-dependent, with most studies representing controlled experimental systems rather than field-ready technologies.

## 5. Toxicity, Safety, and Regulatory Considerations

The rapid expansion of nanoparticle applications in plant science, biotechnology, and cryopreservation increases the need for rigorous evaluation of toxicity, long-term safety, and regulatory implications. Although many nanoparticles provide measurable physiological or technological benefits at low concentrations, their biological reactivity, persistence, and interactions with plant tissues and culture media raise important questions regarding species-specific responses, exposure-dependent outcomes, and biosafety in germplasm conservation and agricultural deployment [164,165]. A responsible integration of nanotechnology into plant research and production systems therefore, requires a clear link between intended use, likely exposure routes, and the most relevant safety endpoints.

Risk interpretation in this field is strongly dependent on application context because exposure pathways and affected receptors differ between open environmental systems and contained laboratory systems. In open field and soil-based uses, including seed coatings, foliar sprays, and soil amendments, release to environmental compartments is inherent to the application, and risk assessment must address environmental fate, transport, transformation, and nontarget impacts across soil, aquatic, and food chain receptors [16,166]. In contrast, in vitro culture platforms and cryobanks are operationally contained settings in which the dominant hazards are associated with worker exposure during nanoparticle handling, management of nanoparticle-containing waste streams, and evaluation of potential nanoparticle persistence or carryover in treated tissues rather than ecosystem-scale exposure [167,168]. This distinction provides a practical framework for aligning safety assessment and mitigation measures with the relevant use scenario.

### 5.1. Plant Toxicity and Degradation

A primary determinant of nanoparticle behavior in plant systems is dose, because many nanomaterials show biphasic dose response patterns in which low concentrations can stimulate growth or stress tolerance, while higher concentrations inhibit growth and activate toxicity pathways [169]. Such patterns emphasize the need to optimize concentration, exposure duration, and delivery method in a species and stage-specific manner, particularly when transitioning from controlled environments to production-scale settings.

At low concentrations, multiple classes of nanoparticles, including silver, zinc oxide, silica, iron oxides, and carbon-based nanomaterials, have been reported to improve germination, enhance root and shoot development, increase photosynthetic pigment content, and activate antioxidant defenses or stress-related pathways. These effects are commonly interpreted as modulation of redox signaling, changes in micronutrient availability, or activation of developmental programs, although the dominant mechanism depends on particle chemistry and transformation behavior in the exposure medium [164,170]. Nevertheless, positive responses observed in controlled assays should not be interpreted as evidence of universal safety. Plant outcomes depend on the balance between signaling-level oxidative cues and damaging oxidative stress.

At elevated concentrations, nanoparticles can induce inhibitory effects including suppression of root and shoot growth, disruption of meristematic activity and cell division, electrolyte leakage, membrane damage, excessive accumulation of reactive oxygen species, and impaired photosynthetic efficiency. Genotoxic endpoints have also been reported in plant test systems, including chromosomal abnormalities and DNA damage under sufficiently high exposures, which supports the use of cytogenetic screening when developing nano-enabled inputs that may contact meristematic tissues or reproductive structures [171,172]. For silver nanoparticles, substantial evidence links phytotoxicity to oxidative stress and to the contribution of released silver species, with outcomes modulated by particle size, surface chemistry, and exposure conditions [164]. For metal oxide systems, toxicity is often influenced by dissolution, surface reactivity, and interactions with plant antioxidants and membranes, which can vary across species and developmental stages.

The fate of nanoparticles after entry into plant systems is a second critical component of safety because accumulation, localization, transformation, and degradation determine both efficacy and potential for delayed effects. Nanoparticle behavior in planta is governed by physicochemical attributes such as size, morphology, surface charge, solubility, and functionalization, combined with biological factors including tissue permeability, metabolic activity, and the presence of endogenous biomolecules that can form coronas and alter reactivity. Environmental parameters such as pH, light exposure, and ionic strength can further influence nanoparticle stability and speciation during plant exposure, particularly in hydroponic systems and culture media.

Persistence and degradability vary substantially by composition. Many metallic and metal oxide nanoparticles are relatively stable and may persist in tissues over extended periods, especially under low metabolic activity conditions, which is relevant for slow-growing in vitro cultures and cryopreserved materials. Persistent particles can contribute to chronic stress signatures, alter metabolic profiles, or interfere with signaling processes if retained near sensitive tissues such as shoot apices [150,164]. In contrast, biopolymeric carriers and some carbon-based nanomaterials can show partial biodegradation or transformation depending on functionalization and enzymatic accessibility, and such behaviors may reduce long-term accumulation risk if degradation products are biologically compatible [170,173].

From a risk management perspective, plant toxicity and degradation should be evaluated as a coupled problem. It is not sufficient to measure only early growth outcomes, because delayed effects can arise from persistent tissue retention or from transformation into more bioavailable species. Therefore, responsible application requires experimental designs that include concentration response characterization, appropriate controls for dissolved metal species where relevant, tissue localization studies, and post exposure monitoring that extends beyond the initial treatment window, particularly for applications involving meristematic tissues, regeneration systems, or germplasm conservation.

### 5.2. Environmental Safety in Open Field and Soil-Based Applications

Nanomaterials used in open field crop systems can enter soil and water compartments through seed coatings, foliar sprays, followed by wash off, incorporation into soil matrices, and mobilization via runoff and drainage. In these scenarios, environmental exposure is intrinsic to the technology, and evaluation must incorporate realistic fate and transport behavior in heterogeneous soils and waters [16,166]. Processes such as aggregation, dissolution, sulfidation, and binding to soil organic matter strongly influence mobility and bioavailability and therefore determine whether nanoparticles remain near application sites or migrate into deeper soil layers and connected aquatic systems.

Experimental evidence indicates that soil–plant systems can exhibit distinct behavior for particulate and dissolved fractions, which supports monitoring frameworks that quantify both forms, especially for partially soluble nanomaterials. For silver systems, mobility and bioavailability depend on soil properties and on particle speciation, with measurable differences between nanoparticle forms and ionic inputs under comparable conditions [174]. Such findings justify the integration of soil-specific chemistry and transformation measurements when extrapolating from laboratory assays to environmental exposure scenarios.

A consistent concern for open systems is the sensitivity of soil microbial functions that underpin nutrient cycling. Studies demonstrate that silver nanoparticles can reduce soil enzyme activities and that outcomes are influenced by soil organic matter and aging processes [175]. Coating chemistry is also mechanistically important because it modulates dissolution and toxicity to nitrifying bacteria, indicating that particle surface design must be considered explicitly when forecasting ecological impact [176]. Longer term assessments further emphasize that functionalization, concentration, exposure duration, and soil texture shape microbial community responses, supporting the need for chronic exposure studies across representative soil types [177].

Aquatic exposure can occur when nanoparticles enter drainage and surface waters, where they may affect algae, invertebrates, and fish through oxidative stress and tissue injury pathways. For zinc oxide nanoparticles, gill-related oxidative injury has been documented in aquatic organisms, providing a mechanistic basis for including gill and oxidative stress endpoints in aquatic hazard characterization where runoff scenarios are plausible [178,179]. Food chain transport is also relevant in open systems. Trophic transfer of metallic nanoparticles and associated effects have been demonstrated in terrestrial food chain models, supporting the consideration of indirect exposure routes for nontarget organisms [180]. In addition, uptake and accumulation of engineered nanomaterials in edible plants have been recognized for over a decade, although the extent and form of residues depend on material type and exposure conditions [181]. These factors justify residue-oriented monitoring and careful interpretation of plant uptake claims, especially when translating from hydroponic or pot studies to field matrices.

Materials and formulation strategies can reduce environmental burden when they are designed to limit persistence and uncontrolled release of reactive species. Biobased and biodegradable carriers such as lignin-derived nanocarriers offer a pathway to controlled release with improved environmental compatibility, but their safety must still be validated under realistic soil and climate conditions and across relevant microbial and invertebrate endpoints [16,173]. Overall, open system deployment is best supported by multiseason and multisite field evidence that tracks residues in soil and water, evaluates soil and rhizosphere microbial structure and function, and quantifies yield and quality outcomes across pedoclimatic contexts [165].

### 5.3. Biosafety for Contained Systems Including In Vitro Platforms and Cryobanks

Contained systems such as tissue culture laboratories, bioreactors, and cryobanks present a distinct biosafety profile because environmental release is not inherent to the application. In these settings, the dominant hazards relate to occupational exposure during nanoparticle preparation and handling, management of nanoparticle-containing waste streams, and verification of potential nanoparticle persistence or carryover in treated tissues that may later be transferred to greenhouse or field environments. Workplace studies indicate that handling dry nanomaterials in laboratory environments can generate measurable airborne nanoparticle exposure, even when work is conducted inside ventilation devices, which supports structured control approaches and task-based exposure assessment [167]. Consequently, standard operating procedures should prioritize minimizing open handling of dry powders, the use of appropriate ventilated enclosures, and the adoption of validated cleaning methods that avoid resuspension.

Waste governance is a central control point in contained systems because culture media, rinses, and consumables can become nanoparticle-contaminated. The waste management literature emphasizes the risk of uncontrolled release through disposal routes and highlights the need for dedicated collection and disposal practices for nanomaterial-containing wastes [168]. In practice, laboratories should implement labeling and segregation of nanoparticle wastes, define disposal routes aligned with institutional hazardous waste procedures, and document materials and concentrations used, especially when protocols are scaled for routine production.

In vitro plant culture applications often employ nanoparticles for antimicrobial purposes or for the modulation of regeneration and growth. For example, silver nanoparticles have been investigated as functional additives that can enhance regeneration performance in thin cell layer systems and can improve in vitro growth responses in micropropagation contexts, although outcomes depend on genotype, dose, and medium composition [17,69]. These benefits do not eliminate safety considerations, because silver nanoparticle exposure can impose oxidative stress in plantlets under in vitro conditions and may lead to accumulation of silver species in tissues, which supports the inclusion of physiological monitoring and tissue residue checks when feasible [182].

In cryopreservation workflows, oxidative stress is recognized as a key driver of cryoinjury, and nanomaterials have been explored as additives that may improve post-thaw survival by reducing oxidative damage. Single-walled carbon nanotubes have been shown to improve survival and reduce oxidative injury in cryopreservation of embryogenic callus, illustrating the mechanistic relevance of redox modulation in cryobiology [150]. Nanoparticle use during cryopreservation also raises additional questions about persistence under low metabolic conditions and about the need to confirm normal development following recovery. Recent studies examining nanoparticle effects on cryopreserved plant material support routine evaluation of survival, growth, and genetic stability-related endpoints when introducing new nanomaterial additives into cryopreservation protocols [22,153]. Accordingly, a contained systems safety framework should combine occupational controls, controlled waste management, and post-treatment screening, rather than relying on environmental fate models that are more appropriate for open-field applications.

### 5.4. Regulatory Readiness, Governance, and Emerging Tools for Safer Innovation

Regulatory readiness for nanomaterials in plant-related applications depends on a clear definition of the intended use scenario because data requirements differ between open environmental deployment and contained laboratory use. For food and feed relevant contexts, the European Food Safety Authority guidance provides a structured approach for risk assessment of nanomaterials within the food and feed chain, which is relevant for applications that may lead to dietary exposure or residues [183]. However, globally harmonized frameworks for agricultural uses, especially for open-system exposures in complex field matrices, remain limited, which contributes to uncertainty in their translation to practice and in public communication [165,184].

Method standardization is critical because nanoparticle behavior can change during testing, and differences in dispersion, aging, and exposure maintenance can alter hazard conclusions. Regulatory-oriented discussions of nanopesticide assessment highlight the need for reliable characterization of nanomaterial properties and for modeling tools that respect the constraints of legislative frameworks [184]. Computational approaches can support prioritization and design. Quantitative nanostructure activity relationship modeling has been proposed as a strategy for predicting biological effects based on physicochemical descriptors, and machine learning approaches that integrate large datasets have been used to project plant responses and uptake patterns under varying exposure conditions [165,185]. These tools can reduce experimental burden, but they require transparent validation and should complement, not replace, scenario-appropriate experimental evidence.

Safe innovation approaches further strengthen governance by embedding safety considerations at early stages of material design. The Safe by Design concept has been articulated as a comprehensive approach to integrate hazard and exposure considerations during nanomaterial development, supporting the selection of chemistries and formulations with reduced risk profiles [186]. Broader Safe and Sustainable by Design frameworks have been advanced in the European context to integrate safety and sustainability dimensions across the lifecycle of advanced materials, which is relevant for nano-enabled agricultural products because sustainability endpoints include persistence, resource use, and potential distributional impacts [187].

Technological innovation also influences safety governance. Reviews in plant nanoscience highlight the growing role of nanosensors for plant monitoring and precision agriculture, where real-time sensing and data integration can reduce unnecessary inputs and support more targeted interventions [188]. In parallel, nanocarrier strategies have been discussed for the delivery of gene editing systems, with the goal of improving targeting and reducing reliance on viral vectors, although plant-specific translation remains an active area of research and should be evaluated with strong biosafety oversight when deployed [188,189].

Overall, scalable and responsible use of nanotechnology in plant systems requires integration of long-term evidence, scenario-specific risk assessment, transparent governance, and design strategies that anticipate both benefits and plausible harms. Distinguishing open-system environmental exposures from contained-system occupational and waste considerations strengthens both scientific interpretation and regulatory alignment.

## 6. Conclusions

Nanotechnology is rapidly advancing plant biotechnology by providing highly tunable tools for precise biomolecule delivery, redox regulation, membrane stabilization, and improved nutrient utilization. Across genetic transformation, tissue culture, stress mitigation, and cryopreservation, NPs consistently enhance efficiency, tissue viability, and regeneration, particularly in species and tissues that are recalcitrant to conventional approaches.

Despite rapid progress, nanoparticle applications in plant systems remain constrained by dose-dependent responses, limited comparability among studies, and incomplete understanding of nanoparticle transformations in soils, culture media, and cryoprotective solutions. Future work should prioritize standardization of characterization and reporting, including describing nanoparticle properties within the actual exposure matrix and using harmonized biological endpoints to enable comparison across species and laboratories. Mechanistic studies should clearly distinguish intact-particle behavior from nanoparticle-derived ionic effects and link uptake and trafficking to defined molecular and physiological outcomes.

Translation will also require realistic validation beyond short term assays, including multi-environment tests that measure persistence, accumulation in edible tissues, and impacts on beneficial microbiomes. Safer and more sustainable formulations should be advanced through biodegradable or biogenic carriers and application-specific designs rather than one-size-fits-all materials. Finally, the most impactful near-term opportunities include transient and DNA-free delivery for genome editing, reproducible protocols for regenerating recalcitrant species, and optimized nanoparticle-assisted cryopreservation workflows that improve post-thaw recovery while minimizing cellular injury.

## Figures and Tables

**Table 1:** Types of Nanoparticles Used in Plant Systems.

NP Types	Representative Examples	Core Properties	Major Plant Applications	References
Metallic NPs	AgNPs, AuNPs, CuNPs, PtNPs	High reactivity, surface plasmon resonance, antimicrobial	Plant protection/disease management, tissue culture support, physiology modulation, growth promotion and abiotic stress alleviation, biosensing, pollution detection	[23,24,25,26,27,28,29,30]
Metal Oxide NPs	ZnO, TiO_2_, Fe_3_O_4_, Al_2_O_3_, SiO_2_, CeO_2_, CuO	Photocatalytic activity, redox modulation, nutrient supply	Abiotic stress mitigation, photosynthesis enhancement and growth promotion, plant disease management, nanopriming, cryoprotection, heavy metal detoxification	[31,32,33,34,35,36,37,38,39,40,41,42]
Carbon-Based NPs	Carbon nanotubes (CNTs), graphene oxide (GO), fullerenes, and carbon dots	High surface area, cellular penetration, and conductivity	Gene delivery, growth regulation, stress mitigation, photosynthesis and nutrient uptake support, physiological modulation, biosensing	[14,43,44,45,46]
Polymeric NPs	Chitosan NPs (CS NPs), Alginate/pectin/Poly(lactic-co-glycolic acid) (PLGA NPs), Polyethylene glycol (PEG)-based systems, biodegradable co-polymers	Biodegradable, tunable release, low toxicity	Controlled delivery of hormones, PGRs, DNA, nutrient, agrochemicals, protective or functional compounds	[47,48,49,50,51]
Biogenic NPs	Plant-extract-derived NPs, microbe-mediated NPs, seaweed/algal biogenic NPs	Eco-friendly, cost-effective, functionalized naturally	Abiotic stress tolerance enhancement, improved growth and physiological performance, biotic stress management, nutrient use efficiency and growth promotion, enhanced photosynthesis, and metabolic activity	[52,53,54,55,56,57,58]
Hybrid/Composite NPs	Polymer–metal composites, mesoporous silica hybrids, metal–organic frameworks, plasmonic–photocatalytic composites, responsive nanosensors	Multifunctional, stimulus-responsive, enhanced stability	Enhanced nutrient delivery and growth promotion, multifunctional stress alleviation, targeted pathogen defense and disease management, controlled delivery of bioactive molecules	[59,60,61,62,63,64]

**Table 2:** Summary of Nanoparticle-Mediated Transformation Studies in Plants.

Plant Species	NP Types	Cargo Delivered	Delivery Method	NP Concentration	Outcome/Key Findings	Reference
*Arabidopsis thaliana*	Single-walled carbon nanotubes (SWCNTs)	Plasmid DNA (Green fluorescent protein—GFP), siRNA	Leaf infiltration	~1–50 µg/mL	High-efficiency transient expression and potent gene silencing in mature leaves.	[120]
*Nicotiana benthamiana*	SWCNTs (PEI-functionalized SWNTs)	Plasmid DNA (GFP)	Leaf infiltration	5 µg/mL (3:1 SWNT:DNA; 167 ng plasmid)	The SWNT platform enables DNA delivery and protein expression in Nb leaves without transgene integration; effective DNA delivery and strong expression in Nb have been demonstrated.	[114,121]
*Nicotiana tabacum*	Multi-walled carbon nanotubes (MWCNTs)	Plasmid DNA pGreen0029 (YFP (yellow fluorescent protein) reporter + nptII marker)	Leaf infiltration/callus	30 µg/mL	Transient expression of YFP in protoplasts and stable transformation of callus and leaf disks under nptII with the production of regenerant plants on selective medium (50 mg/L kanamycin) are demonstrated.	[122,123]
*Solanum lycopersicum* (tomato)	Carbon-based nanomaterials (incl. helical MWCNTs, long/short MWCNTs, few-layer graphene)	Not applicable	Seed exposure/growth medium supplementation	50 µg/mL (CBNs added to growth medium; tomato seeds/seedlings exposed)	Tomato germination and seedling growth enhanced; helical MWCNTs altered gene expression and upregulated a tomato water channel (aquaporin) gene.	[124]
*Gossypium hirsutum* (cotton)	PEI-functionalized SWCNTs (DNA–PEI–SWNT complexes)	Plasmid DNA (e.g., GFP reporter constructs)	Leaf infiltration	500 ng PEI-SWNT per 100 µL infiltration	Transient reporter expression demonstrated in cotton leaves (platform designed to avoid transgene integration).	[114, 121]
*Oryza sativa* (rice; suspension cells)	MWCNTs (as-received and sonicated preparations)	None (toxicity/interaction study; not DNA delivery)	Suspension cell culture incubation	20 µg/mL final concentration	ROS increased and viability decreased; Transmission electron microscopy showed MWCNTs associated with the cell wall and not intracellular, even at the highest tested concentration.	[125]
*Arabidopsis* T87 cells	MWCNTs	None (toxicity study; no DNA cargo)	Cell culture exposure	10–600 µg/mL	Reduced dry weight, viability, chlorophyll and SOD activity; toxicity increased as agglomerate size decreased.	[126]
*Triticum aestivum* (wheat)	Gold nanoparticles (AuNPs)	CRISPR-associated protein 9 (Cas9) RNPs	Particle bombardment (biolistic) into immature embryos	0.6 µm gold particles, 5 µL added per shot	Efficient editing in bombarded embryos with regenerated mutants; protocol details for coating/delivery provided.	[127]
*Triticum aestivum* (wheat)	Gold nanoparticles (AuNPs)	DNA/RNA/protein	In planta particle bombardment (iPB)	0.3–1.2 µm gold particles are used; DNA is coated onto gold by precipitation	Describes a meristem-targeted bombardment workflow; emphasizes that gold particles can bind biomolecules for delivery.	[128]
*Nicotiana tabacum* cv Petite Havana (tobacco)	Mesoporous silica nanoparticles (MSNs) capped with AuNPs	DNA + small molecules	Incubation with plant cells/uptake-based delivery	100–200 nm MSNs AuNP caps ~10–15 nm on MSN surface; pore size described as ~3 nm (for molecular loading/release).	MSNs act as carriers enabling intracellular delivery and (in the system described) stimulus/triggered release from capped pores.	[129]
*Zea mays* (maize)	Magnetic NPs (MNP)/MNP–DNA complexes	DNA	Pollen magnetofection	At DNA:MNP = 4:1, the reported complex size = 212.4 nm	Reported pollen magnetofection workflow and parameter optimization; (paper claims) recovery of transformants/seeds following pollen treatment.	[130]
*Allium cepa* (onion)	CS NPs (CS/pDNA complex)	Plasmid DNA carrying Thio-60 (thionin)	Transformation protocol described as developed previously; used for onion tissue/seedlings (immersion-style handling in the workflow section)	CS/pDNA complex prepared with 0.08% chitosan (25 mM acetic acid, pH 5.5). Particle size = 173.3 nm	Transgenic onion lines expressing thionin; reported increased resistance against *Aspergillus niger* infection.	[131]

**Table 3:** Nanoparticle applications in plant cryopreservation.

Plant Species/Tissue	NP Types	NP Concentration	Cryopreservation Method/NP Application	Main Effects/Outcomes	Reference
*Agapanthus praecox* callus	Carbon nanomaterials: Single-Walled CNTs (diameter 1 nm, length 1 μm), Graphene (diameter 2–10 μm, 95% monolayer oxidized), C_60_, GQDs (graphene quantum dots, diameter 3–5 nm, thickness 1 nm)	0.1, 0.3, and 0.5 g/L/0.3 g/L (optimal)	Vitrification/Carbon nanomaterials added to plant vitrification solution 2 (PVS2)	Better preservation of cellular structures with the presence of SWCNT and C_60_ particles inside callus cells. C_60_ increased survival by 159% compared to untreated controls and decreased the malondialdehyde and H_2_O_2_ contents. The distribution of SWCNTs around cell walls and of C_60_ in mitochondria.	[152]
*Agapanthus praecox* embryogenic callus	SWCNTs	0.1 g/L	Vitrification/SWCNTs added to PVS2 during dehydration	Markedly increased survival after LN; balanced ROS and lipid peroxidation; enhanced activities of antioxidant enzymes; SWCNTs mainly localized near cell wall/vesicles and removed during dilution.	[150]
*Lamprocapnos spectabilis* ‘Valentine’ shoot tips	AuNPs	10, 20, or 30 ppm/10 ppm (optimal)	Encapsulation–vitrification/AuNPs incorporated into alginate bead matrix before plant vitrification solution 3 (PVS3)	Improved recovery of LN-stored shoot tips vs. NP-free control; better growth, metabolic and genetic stability; first report of AuNPs in plant cryopreservation.	[153]
*Lamprocapnos spectabilis* Fukuhara ‘Gold Heart’ and ‘Valentine’ shoot tips	AuNPs, AgNPs, ZnO NP	5 and 15 ppm/5 ppm (optimal)	Encapsulation–vitrification with PVS3/NPs in the preculture medium or alginate beads enriched with different NPs	Selected NP treatments improved post-LN recovery and reduced stress markers, enhanced ex vitro growth and development; cultivar-specific effects on survival, morphogenesis, biochemical markers and stress indicators.	[22]
*Lamprocapnos spectabilis* Fukuhara ‘Gold Heart’ and ‘Valentine’ shoot tips	AuNPs (diameter 6 nm), AgNPs (diameter 6 nm), ZnO NPs (diameter 25 nm),	5 and 15 ppm/5 ppm (optimal)	Encapsulation–vitrification with PVS3/NPs in the preculture medium or alginate beads enriched with different NPs	Influenced the metabolic profile, particularly affecting the synthesis of phenolic acids and aldehydes, as well as the antioxidant mechanisms. Cultivar-specific and NP-dependent effects on the metabolic, structural, and genetic stability.	[151]

## Data Availability

No new data were created or analyzed in this study.

## Abbreviations

The following abbreviations are used in this manuscript:

	General Nanotechnology and Materials
NP	Nanoparticle
NPs	Nanoparticles
AgNPs	Silver nanoparticles
AuNPs	Gold nanoparticles
CuNPs	Copper nanoparticles
PtNPs	Platinum nanoparticles
ZnO NPs	Zinc oxide nanoparticles
TiO_2_ NPs	Titanium dioxide nanoparticles
Fe_3_O_4_ NPs	Iron oxide (magnetite) nanoparticles
Al_2_O_3_ NPs	Aluminum oxide nanoparticles
SiO_2_ NPs	Silicon dioxide nanoparticles
CeO_2_ NPs	Cerium oxide nanoparticles
CNTs	Carbon nanotubes
SWCNTs	Single-walled carbon nanotubes
MWCNTs	Multi-walled carbon nanotubes
GO	Graphene oxide
CQDs	Carbon quantum dots
GQDs	Graphene quantum dots
C_60_	Fullerene (buckminsterfullerene)
	Polymeric and Biogenic Nanocarriers
CS NPs	Chitosan nanoparticles
PLGA	Poly(lactic-co-glycolic acid)
PEG	Polyethylene glycol
MSNs	Mesoporous silica nanoparticles
	Plant Biotechnology and Cell Biology
ROS	Reactive oxygen species
LN	Liquid nitrogen
PVS2/PVS3	Plant vitrification solution 2/3
SOD	Superoxide dismutase
CAT	Catalase
POD	Peroxidase
	Genetic Engineering and Molecular Biology
DNA	Deoxyribonucleic acid
RNA	Ribonucleic acid
siRNA	Small interfering RNA
sgRNA	Single guide RNA
CRISPR	Clustered regularly interspaced short palindromic repeats
Cas9	CRISPR-associated protein 9
RNP	Ribonucleoprotein
GFP	Green fluorescent protein
YFP	Yellow fluorescent protein
	Delivery Techniques and Experimental Methods
iPB	In planta particle bombardment
MNPs	Magnetic nanoparticles
